# Rapid Recovery of an Urban Remnant Reptile Community following Summer Wildfire

**DOI:** 10.1371/journal.pone.0127925

**Published:** 2015-05-20

**Authors:** Robert A. Davis, Tim S. Doherty

**Affiliations:** 1 School of Natural Sciences, Edith Cowan University, Joondalup, Australia; 2 School of Animal Biology, The University of Western Australia, Crawley, Australia; Universidad de Granada, SPAIN

## Abstract

Reptiles in urban remnants are threatened with extinction by increased fire frequency, habitat fragmentation caused by urban development, and competition and predation from exotic species. Understanding how urban reptiles respond to and recover from such disturbances is key to their conservation. We monitored the recovery of an urban reptile community for five years following a summer wildfire at Kings Park in Perth, Western Australia, using pitfall trapping at five burnt and five unburnt sites. The reptile community recovered rapidly following the fire. Unburnt sites initially had higher species richness and total abundance, but burnt sites rapidly converged, recording a similar total abundance to unburnt areas within two years, and a similar richness within three years. The leaf-litter inhabiting skink *Hemiergis quadrilineata* was strongly associated with longer unburnt sites and may be responding to the loss of leaf litter following the fire. Six rarely-captured species were also strongly associated with unburnt areas and were rarely or never recorded at burnt sites, whereas two other rarely-captured species were associated with burnt sites. We also found that one lizard species, *Ctenotus fallens*, had a smaller average body length in burnt sites compared to unburnt sites for four out of the five years of monitoring. Our study indicates that fire management that homogenises large areas of habitat through frequent burning may threaten some species due to their preference for longer unburnt habitat. Careful management of fire may be needed to maximise habitat suitability within the urban landscape.

## Introduction

Urban residential areas are growing at an increasing rate globally and it is estimated that by 2030 urban land cover will increase by more than 1.2 million km^2^ [1]. The global trend towards open urban green space planted with non-native plants and managed for human aesthetic values is resulting in homogenization of habitats and consequent reduction in urban biodiversity [2]. Urbanisation leads to large changes in the composition of native plant and animal communities [3,4] and global estimates have found that urban areas retain on average only 8% of their original bird fauna and 25% of their plant species in remnant native vegetation patches (urban remnants) [5].

For fauna in urban remnants, the primary drivers of extinction are habitat loss, alteration and fragmentation, competition from introduced species, and genetic effects that reduce population viability [3]. In urban and peri-urban environments, urban remnants often become highly fragmented with a low degree of connectivity due to the barrier effect of roads or high density development and this can negatively impact fauna populations [6–8]. Increased isolation of habitats and disrupted dispersal ability can have genetic consequences for fauna, including reduced gene flow [9], inbreeding depression [10] and increased risk of local extinction from stochastic events such as fire or disease [11].

Due to the limited dispersal capacity of many reptile species, their often small home ranges and sensitivity to changes in microhabitat and structural habitat elements [12,13], reptiles in urban remnants can be particularly sensitive to disturbance events such as fire [14,15]. Consequently, anthropogenic impacts that lead to habitat change, including fire, are likely to have significant impacts on reptile communities in urban remnants, where fire frequency is often higher than in intact landscapes [16,17]. Despite this, reptiles have been poorly studied in urban environments and Gardner *et al*. [18] highlighted the need for more studies on reptile responses to habitat alteration, in order to better understand and mitigate regional and large-scale population declines.

Wildfire and prescribed burning typically have strong effects on reptile communities [19,20]. Potential effects include animal mortality [21,22], loss of shelter [23], altered resource availability [24] and changes in thermoregulatory opportunities [25], ultimately leading to changes in the population densities of certain species and associated changes in community composition [26–29]. Habitat suitability is expected to change as vegetation recovers post-fire and certain seral stages often support distinct reptile assemblages [26,30]. Understanding the impact of fire on reptile communities and their habitat is essential for effective conservation management [31].

In this study, we tracked the recovery of an urban reptile community following a summer wildfire in Kings Park, a large urban remnant in Perth, Western Australia. Urban bushland remnants in Perth support diverse reptile communities, with areas as small as a single hectare able to maintain viable populations of some species [32,33]. These remnants are vitally important habitats for reptile assemblages on the surrounding Swan Coastal Plain, particularly in the face of rapid urban expansion [32,33]. We specifically sought to examine how reptile species richness, abundance, body size and community composition differed between burnt and unburnt sites over a period of five years post-fire.

## Methods

### Study Area

**Fig 1:**
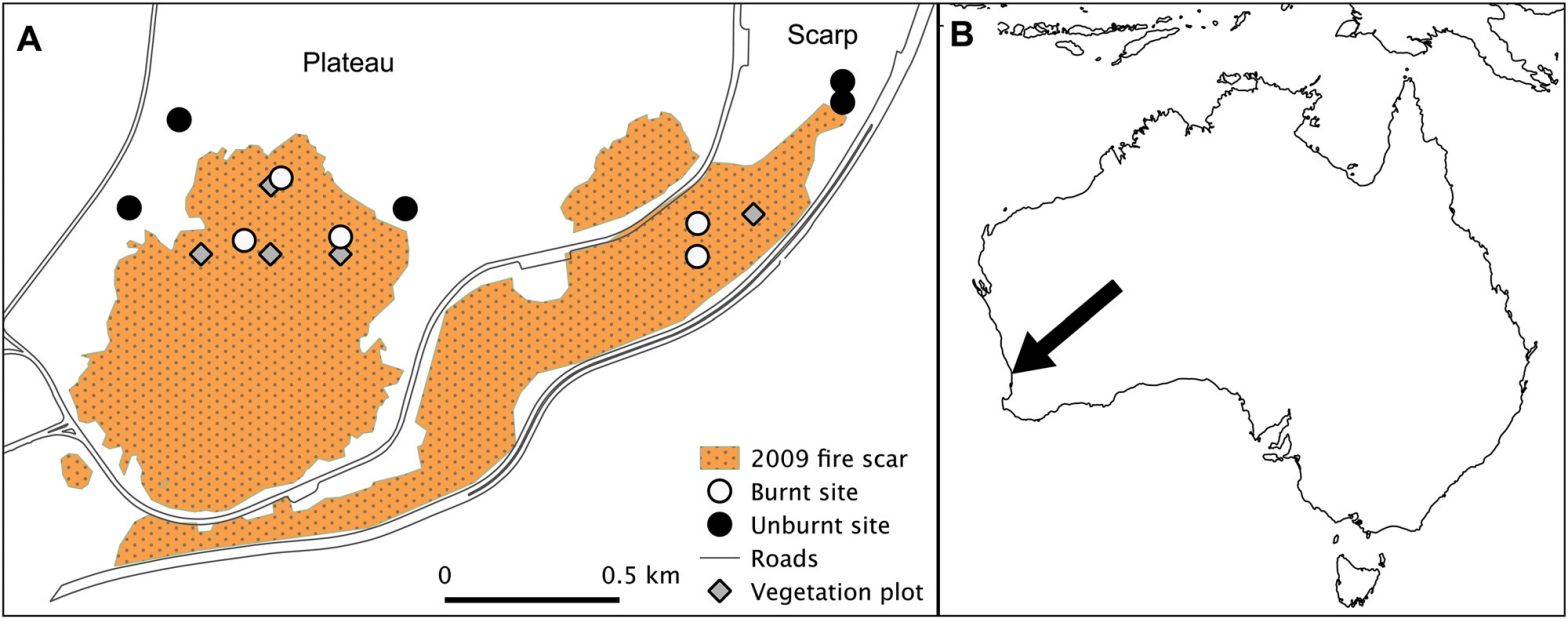
Location of monitoring sites at Kings Park (A) and study site location in south-western Australia (B).

Kings Park is located approximately 1.5 km from the central business district of Perth, Western Australia (31°57’39” S, 115°49’56” E; Fig 1). The park contains 267 ha of mixed *Banksia*, *Allocasuarina*, and *Eucalyptus* spp. woodland alongside a smaller section of botanic gardens [34]. This woodland portion of the park is remnant native vegetation and constitutes a large urban remnant. The majority of the park sits on a plateau that gives way to a steep limestone escarpment on its eastern boundary leading down to the Swan River. The climate is Mediterranean with mild, wet winters and hot, dry summers. Mean annual rainfall is 736 mm, with around 80% falling between May and September inclusive (Perth Metro weather station) [35]. The park has a long history of arson, wildfire and prescribed burning, with an average of 10 fires occurring each year in the period 1944 to 2010 (range 0 to 35) [36]. Between 1963 and 1994, an average of 33.28 ha was burnt each year by wildfire, arson and prescribed burning [37]. Most fires in the park are small (< 1 ha) and since 1944, there have only been nine fires greater than 25 ha in size (< 2% of all fires) [36]. In January 2009, a wildfire burnt 40 ha of bushland on the scarp and plateau at Kings Park (Fig 1). This provided an opportunity to survey the immediate post-fire dynamics of the reptile community.

### Sampling

We established five reptile-monitoring grids in the burnt area and five in older unburnt vegetation. Three of the unburnt sites were last burnt in summer 1988 and the remaining two were last burnt in summer 1978. Due to the small number of replicates in each of these older fire ages, we grouped all of those sites as ‘unburnt’ for the analyses. All sites were monitored for 14 days every austral spring (November) for five years following the fire (2009–2013).

All sites were placed as far apart as possible (50 to 500 m) within the constraints of the fire scar and other logistical constraints within the park. Ground-dwelling reptiles at each site were sampled using nine pitfall traps set up in a 3 × 3 grid, with each trap 10 m apart. The pitfall traps were white PVC buckets 40 cm deep and 30 cm in diameter. Each trap was located in the centre of a 7 m long, 20 cm tall drift fence running in a random direction. Five funnel traps were placed alongside the drift fences of five random pitfall traps at each site. Research was approved by the ECU Animal Ethics Committee under project approval number 4064 from 2009–2014. Trapping was undertaken under a fauna licence granted by the Department of Parks and Wildlife and permission to work in Kings Park (31°57’39” S, 115°49’56” E) was provided by Mr Steve Eason (Botanic Gardens and Parks Authority). No animals were sacrificed and no threatened species were captured or studied. All animals were captured using pitfall traps and funnel traps that were checked very early each morning. Captured animals were measured and released at the point of capture. The pitfall trapping data is available in S1 Table.

### Habitat assessment

We used vegetation data collected as part of a separate study to document post-fire changes in litter cover and bare ground. On the plateau, four 1 × 1 m plots located on a larger 100 × 100 m grid were monitored in 2008, 2009, 2011, 2012 and 2014 (Fig 1). On the scarp, 15 1 × 1 m plots nested within a 10 × 10 m plot were monitored in 2009, 2011 and 2013. All plots were within the 2009 fire scar and close to our trapping sites (Fig 1). Bare ground and litter cover were estimated using modified Domin [38] cover classes: 1–3 (<1% cover), 4 (1–3%), 5 (4–10%), 6 (11–25%), 7 (26–33%), 8 (34–50%), 9 (51–75%), 10 (76–90%) and 11 (91–100%). Total % cover was estimated by summing the midpoint value of each cover class for bare ground and litter cover. To account for the differing sampling methods and to aid visual interpretation, we range-standardised values between 0 (no cover) and 1 (total cover). Data from unburnt areas were not available for comparison.

### Statistical analysis

We used generalised linear mixed models to test the effects of fire on reptile abundance, species richness, diversity and evenness. For each year, we calculated abundance as the number of unique individuals of each species caught at each site. We tested those species for which at least 30 animals were captured across the entire study (i.e. *Ctenotus fallens*, *Hemiergis quadrilineata*, *Pogona minor*, *Cryptoblepharus buchananii*, *Lerista elegans*, *Lerista praepedita* and *Morethia obscura*). We calculated total reptile abundance by summing the number of individuals of all reptile species caught at a site. We calculated Simpson’s diversity index [39] and Pielou’s evenness index for each site and year based on all species. The predictor variables in the models were year (five levels), fire (burnt or unburnt) and the interaction term. We used Wald chi-squared tests at α = 0.05 to determine the significance of main effects and the interaction term. Site was included as a random intercept to account for repeat sampling over time. We used a Poisson error distribution for those variables measured as counts (abundance and richness) and a normal error distribution for non-count data. The Poisson models for some species exhibited symptoms of non-convergence (i.e. very large standard errors and *P* values) because those species were never captured in some combinations of the two factors: fire and year, i.e. in some cases the variables were perfectly collinear [40,41]. To counter this, we fitted the Poisson models using a weakly informative Bayesian prior on the fixed effects in the R package blme [42,43].

We performed a similar set of tests on the mean snout-vent lengths (SVL) of the four most common species (*Ctenotus fallens*, *Hemiergis quadrilineata*, *Cryptoblepharus buchananii* and *Pogona minor*) to determine if there were differences in reptile body size between burnt/unburnt areas and years. Analyses were conducted in r version 3.0.2 [44]. We used general linear models to determine if there were differences in litter cover and bare ground between years. If the main effect of year was significant, we made pairwise comparisons between years using Tukey post-hoc tests. We also present graphs of changes in these variables over time

We used permutational analysis of variance (PERMANOVA) to test for differences in the reptile community between burnt and unburnt sites. In primer version 6.1.12 and permanova+ version 1.0.2 [45], we created a Bray-Curtis resemblance matrix based on species abundance at every site and year. Rare species (caught less than five times) were excluded (*Ctenotus australis*, *Varanus tristis* and *Neelaps bimaculatus*). We fitted a PERMANOVA model using 9999 permutations with main effects of fire and year, an interaction term, and a random effect of site nested within fire. Significance was set at α = 0.05. Following a significant main effects test, we made pairwise comparisons between burnt/unburnt within each year and recorded average percentage similarity between burnt/unburnt sites for each year. We calculated an additional resemblance matrix using the ‘distance from centroids’ function and plotted these results using non-metric multidimensional scaling (MDS) to visualise compositional changes over time.

## Results

Across the entire study, we captured 1061 individual reptiles from 19 species (skinks: 11 species; legless lizards: 2; elapid snakes: 2; blind snakes: 1; geckoes; 1; agamids: 1; varanids: 1; see S1 Table). The most commonly captured species were the skinks *Ctenotus fallens* (n = 464), *Hemiergis quadrilineata* (n = 181), *Cryptoblepharus buchananii* (n = 62), *Lerista elegans* (n = 52), *L*. *praepedita* (n = 61) and *Morethia obscura* (n = 36), and the agamid *Pogona minor* (n = 66).

**Fig 2:**
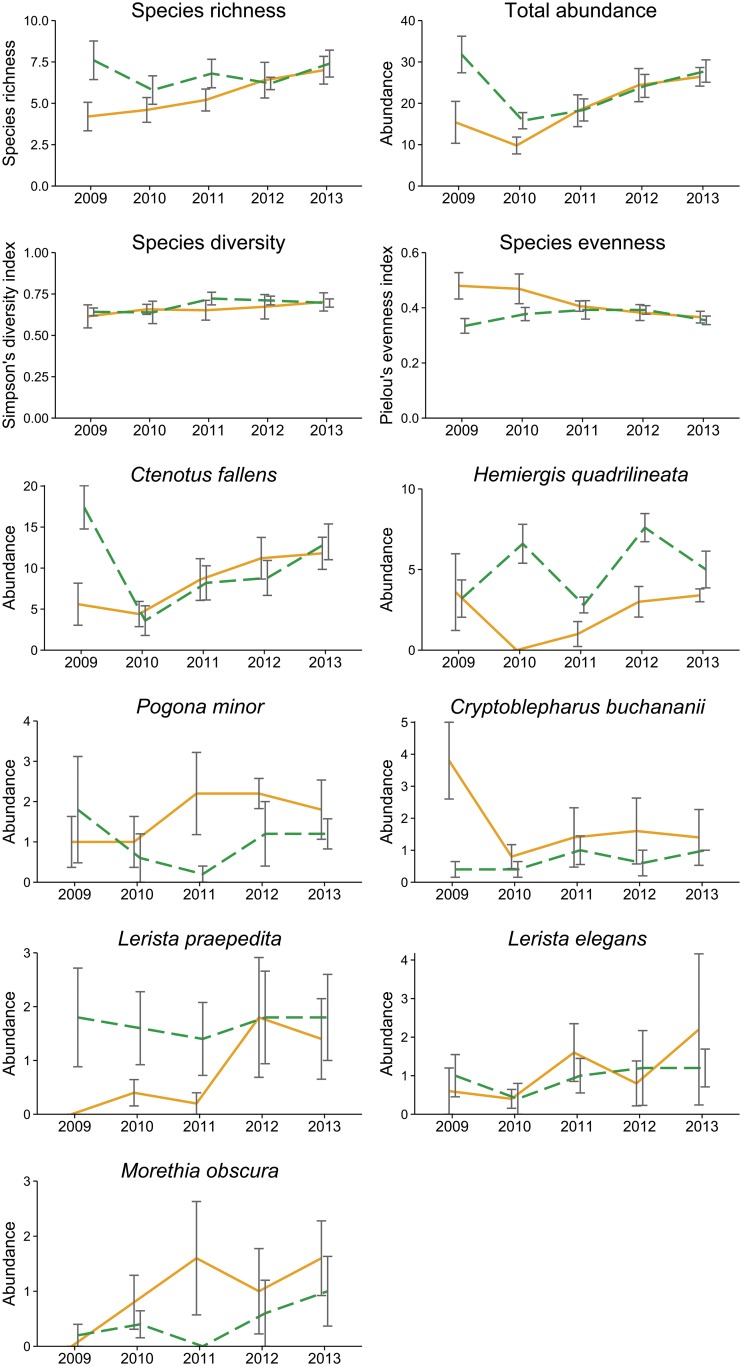
Mean and standard error of reptile abundance, species richness, diversity and evenness at burnt (solid orange line) and unburnt (dashed green line) sites in each year.

**Fig 3:**
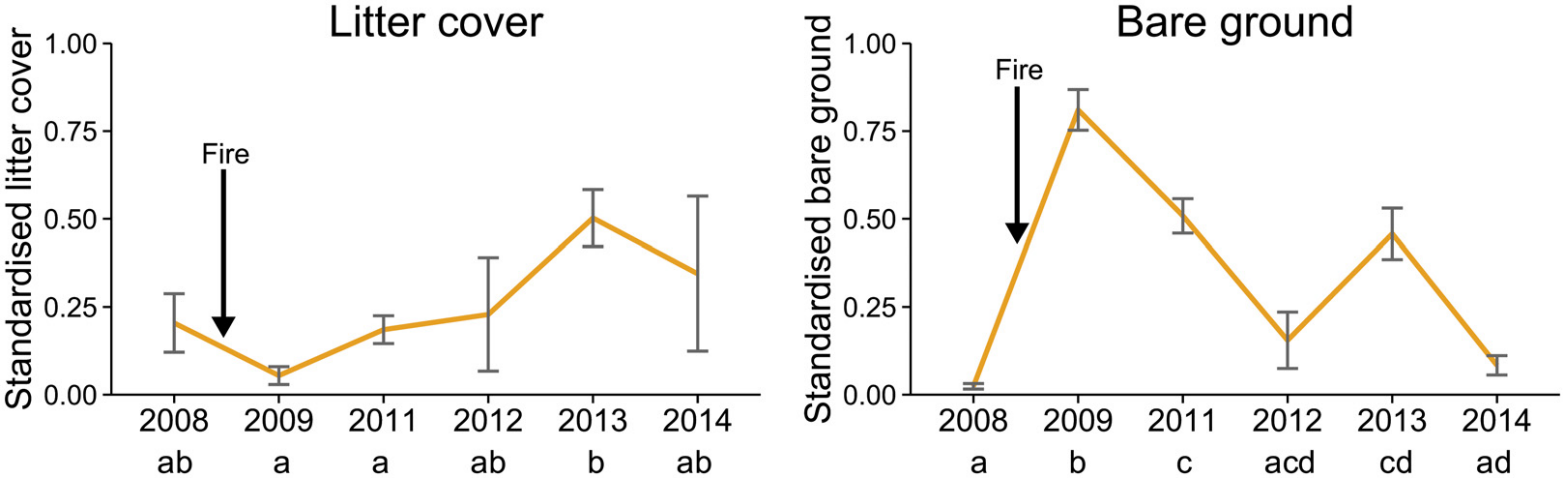
Means and standard errors for bare ground and litter cover in vegetation plots within the fire scar at Kings Park. The fire occurred in January 2009, so the measurements from 2008 are ‘pre-fire’. Pairwise differences are indicated using subscripts, with significantly different pairs not sharing the same letter.

There was a significant interaction between year and fire for four of the response variables, significant fire effects for five variables, and significant year effects for five variables (Table 1). Reptile species richness at unburnt sites was higher than that at burnt sites for the first three years post-fire, with no difference between burnt and unburnt sites in the final two years (Fig 2), although the interaction between fire and year was not statistically significant (Table 1). Total reptile abundance at unburnt sites was higher than at burnt sites in the first two years following the fire, but was similar between burnt and unburnt sites after that (Fig 2). Conversely, species evenness at burnt sites was higher than unburnt sites for the first two years (Fig 2). *Ctenotus fallens* abundance at unburnt sites was more than three times higher than burnt sites in the first year, but was similar between the two in all other years (Fig 2). *Hemiergis quadrilineata* abundance was higher in unburnt sites in all years except the first (Fig 2). *Cryptoblepharus buchananii* abundance was higher at burnt sites in the first year only, although the interaction between fire and year was not statistically significant (Table 1). Species diversity, and the abundance of *Pogona minor*, *L*. *praepedita*, *Lerista elegans* and *Morethia obscura* did not show clear responses to fire or year (Fig 2, Table 1). There was a significant effect of year on litter cover (*χ* ^2^ = 33.93 (5), *P <* 0.001) and bare ground (*χ* ^2^ = 73.00 (5), *P <* 0.001) at vegetation monitoring plots within the fire scar. The amount of bare ground at burnt sites spiked immediately following the fire and decreased throughout the study, whereas litter cover decreased immediately following the fire and then increased throughout the remainder of the study (Fig 3).

**Table 1:** Wald chi-squared tests for the effect of fire and year on reptile richness, abundance, evenness and diversity.

Response variable		Year _(4)_	Fire _(1)_	Year × Fire _(4)_
Reptile richness	χ² =	4.74	**4.26**	2.98
	*P =*	0.315	**0.039**	0.56
Total reptile abundance	χ² =	**45.49**	**12.00**	**21.65**
	*P =*	**<0.001**	**<0.001**	**0.001**
Diversity	χ² =	1.74	0.14	1.09
	*P =*	0.784	0.706	0.896
Evenness	χ² =	**16.35**	**10.87**	**13.46**
	*P =*	**0.003**	**<0.001**	**0.009**
*Ctenotus fallens*	χ² =	**23.65**	**11.24**	**24.32**
	*P =*	**<0.001**	**<0.001**	**<0.001**
*Hemiergis quadrilineata*	χ² =	**14.61**	0.31	**13.09**
	*P =*	**0.006**	0.576	**0.011**
*Pogona minor*	χ² =	3.27	0.15	5.03
	*P =*	0.514	0.700	0.285
*Cryptoblepharus buchananii*	χ² =	**9.95**	**5.14**	3.15
	*P =*	**0.041**	**0.023**	0.532
*Lerista elegans*	χ² =	7.72	0.28	1.92
	*P =*	0.102	0.594	0.750
*Lerista praepedita*	χ² =	8.95	2.26	5.23
	*P =*	0.062	0.133	0.264
*Morethia obscura*	χ² =	4.63	0.08	1.97
	*P =*	0.328	0.777	0.741

Significant terms are indicated with bold text. Degrees of freedom are indicated using subscript in the column headings.

We captured 12 additional species too infrequently to perform statistical analyses on (*n =* 1–25 total animals captured). However, six of these rarely caught species (*Ctenotus australis*, *Cyclodomorphus celatus*, *Lerista lineopunctulata*, *Lialis burtonis*, *Menetia greyii* and *Ramphotyphlops australis; n* = 2–25 animals) were captured most frequently at unburnt sites (72–100% of captures). A single *Varanus tristis* was also captured at an unburnt site. Two other species, *Tiliqua rugosa* and *Christinus marmoratus*, were captured most frequently at burnt sites (72–87%, *n =* 15–18).

**Fig 4:**
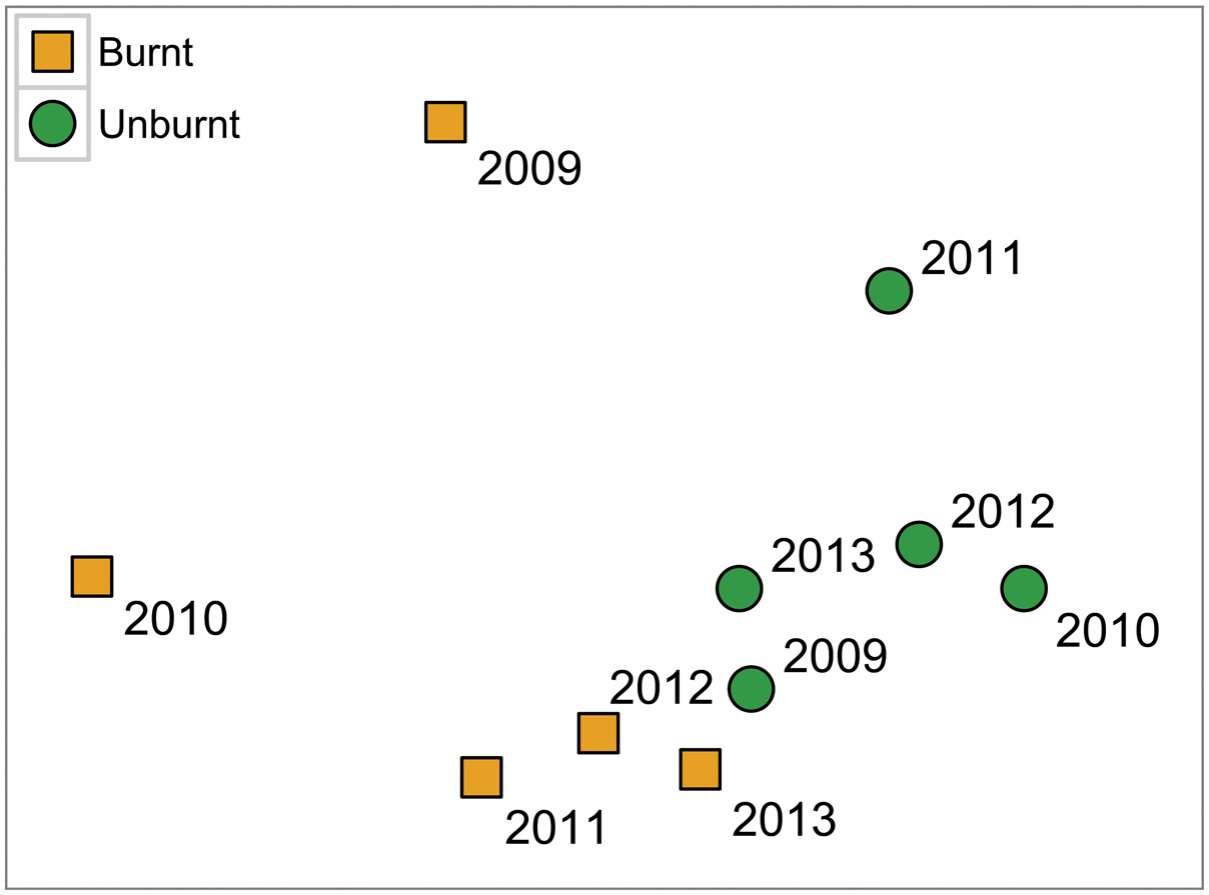
MDS plot of reptile community composition in each sampling year at burnt (square orange symbols) and unburnt sites (round green symbols). Stress = 0.05.

All terms were significant in the PERMANOVA on reptile community composition (Table 2). Pairwise tests showed that community composition was significantly different between burnt and unburnt sites in the first three years, but not the final two (Table 3, Fig 4). Similarity in community composition between burnt and unburnt sites generally increased over the study period, although there was an early decline between 2009 and 2010 (Table 3, Fig 4).

**Table 2:** PERMANOVA results for the effect of fire, year and site on reptile community composition.

Factor	df	Pseudo-*F*	*P*
Fire	1	**2.85**	**0.015**
Year	4	**2.20**	**0.004**
Site	8	**3.72**	**<0.001**
Year × fire	4	**3.11**	**<0.001**

Significant terms are indicated with bold text.

**Table 3:** Pairwise PERMANOVA tests between burnt and unburnt sites in each year.

Year	*t*	*P*	% similarity
2009	**1.78**	**0.024**	**41.61**
2010	**2.07**	**0.007**	**29.24**
2011	**1.78**	**0.006**	**42.17**
2012	1.47	0.086	52.74
2013	0.96	0.511	60.28

Significant terms are indicated with bold text.

**Fig 5:**
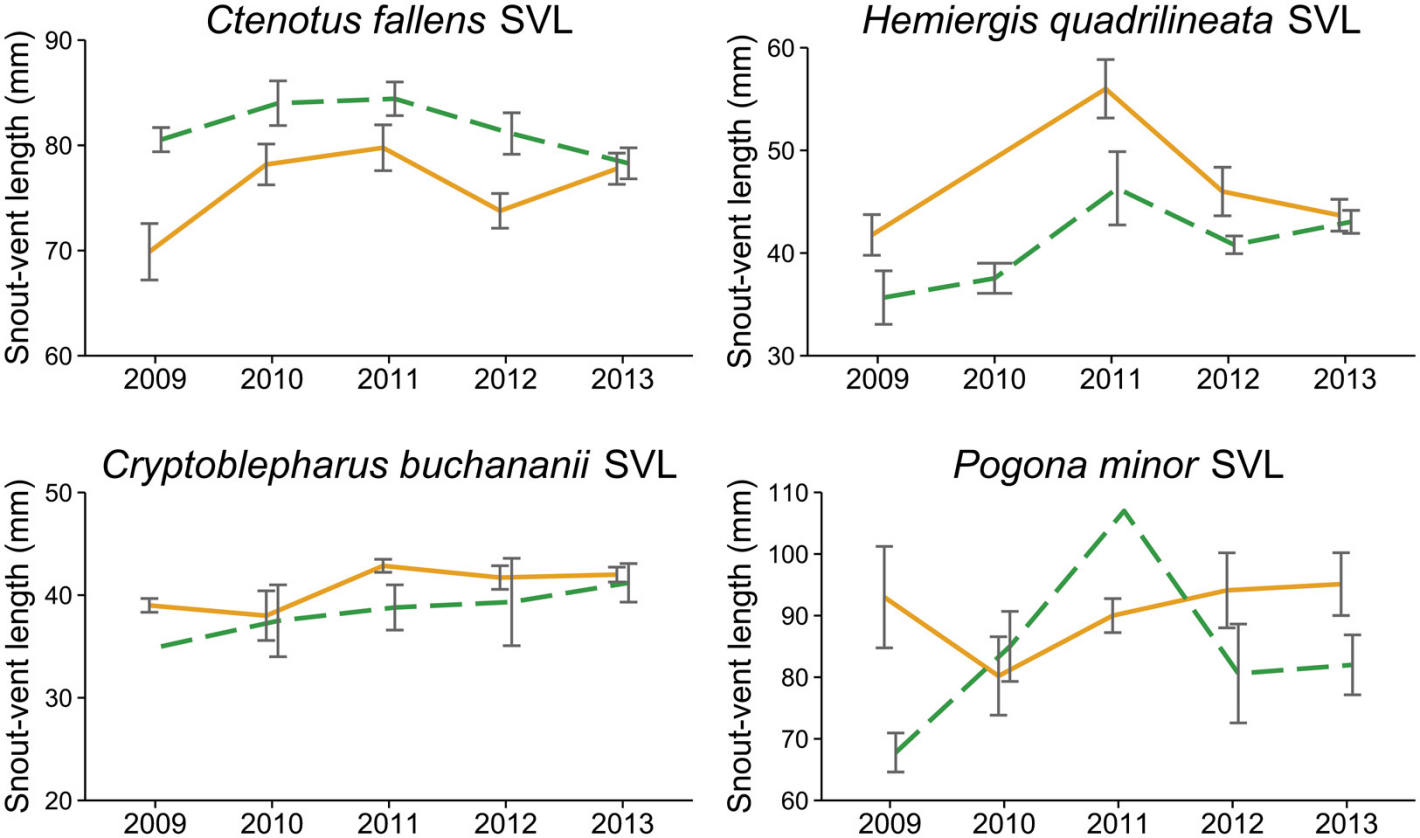
Mean snout-vent length (SVL) for the three most common species at burnt (solid orange line) and unburnt (dashed green line) sites in each year.

There was a significant effect of year × fire on the mean body size (SVL) of *Ctenotus fallens* (Table 4). The main effect of fire was significant for *Ctenotus fallens*, *Hemiergis quadrilineata* and *Pogona minor* (Table 4). The SVLs of *Ctenotus fallens* captured at unburnt sites were higher than those captured at burnt sites in the first four years of the study, but not the fifth (Fig 5). In contrast, the SVLs of *Hemiergis quadrilineata* at burnt sites were higher than those at unburnt sites for the first four years of the study (Fig 5), although the interaction between year and fire was not statistically significant (Table 4). The SVLs of *Cryptoblepharus buchananii* and *Pogona minor* did not show a clear relationship with either year or fire (Fig 5, Table 4).

**Table 4:** Effect of fire and year on mean snout-vent lengths (SVL) for *Ctenotus fallens*, *Hemiergis quadrilineata*, *Cryptoblepharus buchananii* and *Pogona minor*. Significant terms are indicated with bold text. Degrees of freedom are indicated using subscript in the column headings.

Species		Year _(4)_	Fire _(1)_	Year × Fire _(4)_
*Ctenotus fallens* SVL	χ² =	**18.31**	**14.89**	**12.65**
	*P =*	**0.001**	**<0.001**	**0.013**
*Hemiergis quadrilineata* SVL	χ² =	**14.43**	**4.67**	4.83
	*P =*	**0.006**	**0.031**	0.185
*Cryptoblepharus buchananii* SVL	χ² =	7.85	1.09	2.13
	*P =*	0.097	0.297	0.712
*Pogona minor* SVL	χ² =	4.17	**9.93**	9.08
	*P =*	0.384	**0.002**	0.059

## Discussion

### Reptile community response to fire

The relict urban reptile community that we studied recovered rapidly following a summer wildfire. Unburnt sites initially had higher reptile species richness and total abundance, but burnt sites rapidly converged, recording a similar total abundance to unburnt areas within three years, and a similar richness within four years post-fire. Species diversity in our study did not show a clear change as a result of fire. Working at the same site, Dell and How [46] also found a rapid recovery of the reptile community post-fire. In their study, the number of species and the number of individuals in burnt sites had declined markedly in the first year after burning when compared to unburnt sites. In the second and third years, species richness was equal in burnt and unburnt sites and the number of individuals in the burnt site was only marginally lower [46].

In contrast to these findings, a study of a Mediterranean reptile community in France found a loss of reptile diversity after one or multiple fires, as well as a change in species composition favouring insectivorous, open habitat species [47]. This study and many others have suggested that reptiles show a strong successional response to fire that is driven by changes to habitat structure [48], however other studies have found no such successional response to fire, due to the rapid post-fire recovery of vegetation structure [49]. Previous research at our study site found that lizard diversity was highest at sites that had remained unburnt for the longest period of time (up to 15 years), and several species were absent from sites last burnt four to six years prior (*Diplodactylus polyophthalmus*, *Lialis burtonis*, *Lerista elegans*, *L*. *lineopunctulata*, *L*. *praepedita* and *Morethia obscura*), despite being present in longer unburnt areas [46]. Also working in the same region, Valentine *et al*. [50] found that total reptile abundance was significantly higher in longer unburnt areas (>16 years post-fire), although individual species responses varied. Individual responses are likely to be a product of species life history, trophic position, dispersal capacity and habitat preferences, and there is evidence that reptiles reliant on understorey cover and leaf-litter often show more significant short-term responses to fire than other species [49,51].

The rapid recovery of the reptile community recorded here may also have been related to the relatively small size of the fire (40 ha). Specifically, a much larger fire could potentially slow reptile recovery by increasing the area to edge ratio of the burnt area, hence increasing the distance over which dispersing animals must travel to recolonise burnt habitat [52,53]. However, data on the dispersal capacity of most species in this study is lacking. Also, we are not aware of any study that has examined the influence of burned area patch size on the speed of reptile recovery post-fire. Rather than speculating further here, we simply acknowledge that fire size may have influenced the speed of recovery we recorded, and recommend that future studies examine species dispersal ecology and investigate how patch size influences recovery processes.

### Habitat structure and reptile responses

Except for the first year, the fossorial skink *Hemiergis quadrilineata* was most abundant in unburnt sites throughout the study and remained so in the final year. This species may be responding to the loss of leaf litter, coarse woody debris and other ground-cover elements as a result of the fire. Leaf litter cover decreased immediately following the fire and there was more bare ground post-fire than pre-fire. Several studies in our region support the notion of fire-mediated changes to reptile microhabitat in Banksia woodlands. Valentine *et al*. [50] and Burrows and McCaw [54] both found support for the greatest accumulation of ground litter fuel within the first four to six years post-fire, followed by a stable accumulation at six to 20 years post-fire. Valentine *et al*. [50] identified leaf litter cover and depth as one of the most significant explanatory variables for *H*. *quadrilineata* abundance in Banksia woodlands, which may explain why *H*. *quadrilineata* abundance in burnt areas had not reached that of unburnt areas by the conclusion of our study. Other species rarely captured at burnt sites in our study are also known to have positive relationships with leaf litter microhabitat, such as *Lialis burtonis* [55] and *Menetia greyii* [50], thus confirming the role of leaf litter accumulation in providing suitable habitat for several reptile species in Banksia woodlands.

In contrast to the responses of the leaf-litter dwelling species, recently burnt sites may be favoured by some reptile species due to enhanced thermoregulatory opportunities and increased access to prey. Santos and Cheylan [47] found that reptile communities in Mediterranean habitats followed a habitat accommodation model, with recently burnt areas (≤10 years post-fire) being dominated by insectivorous, open-habitat species that were specialists in their niche and had a short life span. Although no species in our study showed a lasting preference for burnt areas, the arboreal skink *Cryptoblepharus buchananii* displayed a strong immediate post-fire preference for burnt areas that may reflect exploitation of some advantage such as increased resources or basking habitat, or the displacement effect noted by Driscoll *et al*. [51]. Many *Cryptoblepharus* species are generalist or disturbance specialists, favouring habitat edges [56] and urban areas [33], and *Cryptoblepharus buchananii* is a very common garden species in Perth, where it basks on walls and fences. Interestingly, despite its generalist nature, Valentine *et al*. [50] found this species to be more strongly associated with long unburnt areas in their study. This is in great contrast to our findings and may be more reflective of the higher residential density surrounding our study site (Valentine *et al*. [50] worked in a continuous wooded landscape), which may result in greater population sizes of this generalist and potentially disturbance tolerant species.

Another species, the skink *Ctenotus fallens*, exhibited an initial peak in abundance in unburnt areas immediately following the fire, followed by a convergence in abundance between burnt and unburnt areas by the second year and all others to follow. Although we have no data on dispersal, this may represent a post-fire influx of individuals emigrating from burnt to unburnt areas, and this intriguing observation warrants further investigation. Interestingly, Valentine *et al*. [50] noted that *C*. *fallens* was associated with older fire ages and did not report any association with early post-fire stages. The post-fire peak we observed, fits well with the suggestion by Driscoll *et al*. [51] that short-term reptile movements occur in response to fires. These likely do not represent actual demographic changes in the population, but rather short-term displacement of individuals seeking refuge or selecting preferred habitat.

### Body size and fire

Interestingly, the body size of adult *Ctenotus fallens* (spring captures) in burnt areas was smaller than those captured in unburnt areas. Although little information is available on this phenomenon, Rodriguez-Caro *et al*. [57] found a negative effect of fire on the growth rate of spur-thighed tortoises in Spain, possibly due to changed food resources. Lower prey availability in burnt areas may explain the smaller body sizes of *C*. *fallens* captured in those areas in our study [58], although these size differences could also be the result of natal dispersal [59], or competitive interactions leading to exclusion from food resources in unburnt areas [60], especially since *C*. *fallens* exhibits aggressive territorial behaviour [61].

### Managing burning regimes for reptiles in urban remnants

Urban reserves, such as our study site, experience high rates of arson and wildfire, hence leading to increases in fire frequency [16,17]. Such increases are likely to reduce the availability of long unburnt habitat, potentially threatening species that are most abundant in these areas [15,30,50]. Given that no species showed a strong preference for recently burnt habitat (≤ 5 years since fire), but unburnt areas had the highest species richness and number of unique species, management at our study site should aim to maintain some extensive areas of long unburnt habitat in the park. Systematic fire mapping and information on species ecology should be used to inform fire management guidelines [31,62] and such strategies must also consider the potentially competing needs of other taxa within the system, such as bird and plant communities.

Given the diversity of responses exhibited by different reptile species, it is difficult to propose fire management recommendations that would meet the needs of all species. Wilson *et al*. [63] recommended that burning regimes for Banksia woodlands in this region should use multiple response variables, including reptile abundance. Consequently, Wilson *et al*. [63] presented an idealised curve of fire ages that incorporated the needs of multiple biotic components. Modelling of existing fire ages indicated that longer unburnt habitats are greatly under-represented and more recent fire ages are greatly over-represented on the northern Swan Coastal Plain. Such effects are likely to be greatly exacerbated when considering highly isolated urban remnants such as our study site. These sites often suffer higher rates of arson than more extensive bushland areas and are also more susceptible to other human impacts [16]. The compounding effects of fire, isolation and human disturbance increase the likelihood of extinction for urban reptile assemblages. Maintaining areas of longer unburnt habitat, in the face of increasing wildfire, should help to ensure the future persistence of a diverse urban reptile assemblage.

## Supporting Information

S1 Dataset Comma separated value file of pitfall trapping data from Kings Park between 2009 and 2013. (CSV)

S1 Table List of reptile species captured at Kings Park, Perth in Western Australia between 2009 and 2013. Taxonomy is arranged by Family and based upon the WA Museum Checklist of Vertebrate Fauna [64].
(DOCX)
