# Supplementary material for: Rapid Recovery of an Urban Remnant Reptile Community following Summer Wildfire
Source: PLoS One. 2015 May 20;10(5):e0127925. doi: 10.1371/journal.pone.0127925 (PMC4439120; doi:10.1371/journal.pone.0127925)
Supplement: S1 Table — Taxonomy is arranged by Family and based upon the WA Museum Checklist of Vertebrate Fauna [64]. (DOCX) [file pone.0127925.s002.docx]

**S1 Table. List of reptile species captured at Kings Park, Perth in Western Australia between 2009 and 2013.** Taxonomy is arranged by Family and based upon the WA Museum Checklist of Vertebrate Fauna [59].

| Species |
| --- |
| **Gekkonidae** (geckos) |
| *Christinus marmoratus* |
|  |
| **Pygopodidae** (legless lizards) |
| *Aprasia repens* |
| *Lialis burtonis* |
|  |
| **Agamidae** (dragons) |
| *Pogona minor* |
|  |
| **Egerniidae** (lizards) |
| *Cyclodomorphus celatus* |
| *Tiliqua rugosa* |
|  |
| **Eugongylidae** (lizards) |
| *Cryptoblepharus buchananii* |
| *Menetia greyi* |
| *Morethia obscura* |
|  |
| **Sphenomorphidae** (lizards) |
| *Ctenotus australis* |
| *Ctenotus fallens* |
| *Hemiergis quadrilineata* |
| *Lerista elegans* |
| *Lerista lineopunctulata* |
| *Lerista praepedita* |
|  |
| **Varanidae** (monitors) |
| *Varanus tristis* |
|  |
| **Typhlopidae** (blind snakes) |
| *Ramphotyphlops australis* |
|  |
| **Elapidae** (venomous snakes) |
| *Pseudonaja affinis* |
| *Neelaps bimaculatus* |
